# Dracocephalum: Novel Anticancer Plant Acting on Liver Cancer Cell Mitochondria

**DOI:** 10.1155/2014/892170

**Published:** 2014-07-17

**Authors:** Mojtaba Talari, Enayatollah Seydi, Ahmad Salimi, Zhaleh Mohsenifar, Mohammad Kamalinejad, Jalal Pourahmad

**Affiliations:** ^1^Department of Pharmacology and Toxicology, Faculty of Pharmacy, Shahid Beheshti University of Medical Sciences, P.O. Box 14155-6153, Tehran, Iran; ^2^Ayatollah Taleghani Educational Hospital, Faculty of Medicine, Shahid Beheshti University of Medical Sciences, P.O. Box 14155-6153, Tehran, Iran

## Abstract

*Dracocephalum kotschyi* Boiss. (*Labiatae*) is a native Iranian medicinal plant which has been used in combination with *Peganum harmala* L. as a remedy for many forms of human cancer especially leukemia and gastrointestinal malignancies. Hepatocellular carcinoma (HCC) is the third leading cause of cancer-related death worldwide. In this investigation HCC was induced by a single intraperitoneal injection of diethylnitrosamine (DEN) in corn oil at 200 mg/kg body weight to rats. Two weeks after DEN administration, cancer development was promoted with dietary 2-acetylaminofluorene (2-AAF) (0.02%, w/w) for 2 weeks. Serum alpha-fetoprotein (AFP) concentration, serum alanine transaminase (ALT), aspartate transaminase (AST), and alkaline phosphatase (ALP) activities were also determined for confirmation of hepatocellular carcinoma induction. Then rat hepatocytes were isolated with collagen perfusion technique and tumoral hepatocytes were sorted by flow cytometry. Finally isolated mitochondria obtained from both tumoral and nontumoral hepatocytes were used for any probable toxic effect of *Dracocephalum kotschyi* ethanolic extract. Our results showed that *D. kotschyi* extract (250 *µ*g/mL) induced reactive oxygen species (ROS) formation, mitochondrial membrane permeabilization (MMP), and mitochondrial swelling and cytochrome c release only in tumoral but not nontumoral hepatocyte. These findings propose *Dracocephalum kotschyi* as a promising candidate for future anticancer research.

## 1. Introduction

HCC is the most common type of liver cancer. Most cases of HCC are secondary to either a viral hepatitis infection (hepatitis B or C) or cirrhosis (alcoholism is the most common cause of hepatic cirrhosis) [1]. More than 600,000 people die from HCC each year. Worldwide research on the disease needs to be intensified in both the medical and pharmaceutical fields. The incidence of this disease is increasing and it is one of the key indications for liver transplantation. A considerable number of antitumor agents currently used in the clinic are of natural origin. Over half of all anticancer prescription drugs approved internationally are natural products or their derivatives [2, 3]. DEN is a chemical carcinogen which causes the cirrhosis in liver and other organs and is widely used to initiate hepatocarcinogenesis in rats while the further promotion of cancer phenotype might be caused by 2-AAF, phenobarbital, carbon tetrachloride, and other chemicals [4].

*D. kotschyi* is a wild-growing flowering plant belonging to the family* Labiatae* and is found abundantly in southwestern Asia.* D. kotschyi* has been used as a medicinal herb for several years in Iran folk medicine due to its antispasmodic and analgesic properties. Antihyperlipidemic [5] and immunomodulatory [6] effects have also been reported for* D. kotschyi*.

As an important organelle in the cells, mitochondria not only play a central role in calcium and energy metabolism [7], but also are essential components of the apoptotic machinery and by themselves are very important sites of reactive ROS generation in tumoral cells [8]. Because of their important functions in energy production and in regulation of cell death, mitochondria have been considered to be a potentially important target for anticancer drug development, and this strategy has recently gained momentum [9]. Interestingly, numerous notable differences in the structure and function of mitochondria between tumoral and normal cells have been reported [10]. For instance, there are various changes in the size, shape, and number of the mitochondria in liver tumoral cells when compared to their corresponding normal cells. It was reported that certain fast growing tumor cells seemed to have fewer and smaller mitochondria than slowly growing tumors, while certain relatively benign tumors (such as oncocytic adenomas) exhibited large numbers of mitochondria and high levels of oxidative enzymes [11]. A prominent metabolic alteration in tumoral cells is that they exhibit a substantial increase in aerobic glucose metabolism and seem to rely more on mitochondrial pathway for generation of ATP and for production of other molecules for cell growth and proliferation. This phenomenon, known as the Warburg effect, has been observed in a variety of cancer types including solid tumors and leukemia [12]. It should be pointed out, however, that the mitochondrial morphological changes and functional alterations observed in one cancer type or cell line should not be generalized as common mitochondrial abnormalities in all tumoral cells. Although mitochondrial dysfunction is often observed in cancer, it is likely that the specific alterations may vary depending on the cancer types, tissue origins, disease stages, proliferation and differentiation states, and microenvironment such as hypoxia.

In this study we used DEN as initiator and 2-AAF as a promoter for induction HCC in Wistar rat. We aimed to investigate the selective effect of* Dracocephalum* on liver cancer cell mitochondria to activate apoptosis signaling.

## 2. Material and Methods

### 2.1. Animals

Male Sprague-Dawley rats (250–300 g), fed a standard chow diet and given water ad libitum, were used in all experiments. They were purchased from Institute Pasteur (Tehran, Iran) and were kept in individual cages in a controlled room temperature (20°C–25°C) and humidity (50%–60%) and exposed to 12 h of daylight. All experiments were conducted according to the ethical standards and protocols approved by the Committee of Animal Experimentation of Shahid Beheshti University of Medical Sciences, Tehran, Iran. All efforts were made to minimize the number of animals used and their suffering.

### 2.2. *Dracocephalum kotschyi* Boiss (*D. kotschyi*)

The aerial parts of* D. kotschyi* were purchased from an herbal drug store in Isfahan province and the authenticity of the specimen was confirmed by Mohammad Kamalinejad, Faculty of Pharmacy, Shahid Beheshti University of Medical Sciences. A voucher specimen (PMP-304) was deposited at the Herbarium of the Faculty of Pharmacy, Shahid Beheshti University of Medical Sciences.

### 2.3. Preparation of the Extract

The aerial parts (1000 g) of* D. kotschyi* were powdered using an electric grinder. We then extracted 250 g of* D. kotschyi* powder with 1500 mL of 50% ethanol and kept the extract overnight. The extract was filtered and dried. Then 0.5 g dried extract was reconstituted in 2 mL DMSO for our mitochondrial experiments [13]. For standardization process the amounts of the eight flavonoids were measured in this extract.

### 2.4. Analysis Detail for the Amounts of the Eight Flavonoids Recovered from* D. kotschyi* Extract

The dried material obtained from plant extraction was subjected to reversed-phase HPLC using an isocratic solvent system consisting of 55% HCl 0.01 M, 25% acetonitrile, 19% methanol, and 1% water. The flow rate was 7 mL/min and the column was maintained at ambient temperature. The injection volume was 1.7 mL and the detector was set at 226 nm. Data acquisition was carried out using an Advantec PCI 1716 data acquisition card and an in-house developed software. Fractions were collected and pooled from different runs and each fraction was weighed after drying in vacuum (see Table 1).

### 2.5. Experimental Design

The experiment was designed to test the effects of* D. kotschyi* on the hepatocarcinogenesis process in male rats. Hepatocarcinogenesis was induced by a single intraperitoneal injection of DEN in corn oil at 200 mg/kg body wt. Two weeks after DEN administration, cancer development was promoted with dietary 2-AAF (0.02%, w/w) daily for 2 weeks. The rats were divided into two groups of ten animals each.

*Group I (Control).* The rats of this group were given standard rat chow and tap water ad libitum.

*Group II (DEN+2-AAF).* The body weight of each rat was recorded before blood was collected by cardiac puncture. The blood samples were allowed to clot before centrifugation at 1000 ×g for 10 min and 4°C to obtain serum. On the 28th day after DEN administration, hepatocytes and mitochondria were isolated from Wistar rat liver Groups I, II and then treated with 250, 500, and 1000 *μ*g/mL of* Dracocephalum* extract [14].

### 2.6. Serum Alpha-Fetoprotein

Serum alpha-fetoprotein (AFP) concentrations were determined using the ADVIA Centaur AFP bioassay (Siemens, Germany) [14].

### 2.7. Liver Function Tests

Serum alanine transaminase (ALT), aspartate transaminase (AST), and alkaline phosphatase (ALP) determinations were done spectrophotometrically using the Hitachi-912 Chemistry Analyser (Mannheim, Germany) and standard diagnostic kits (Roche Diagnostics) [14].

### 2.8. Isolation of Hepatocytes

Hepatocytes were obtained by collagenase perfusion of the liver and viability was assessed by plasma membrane disruption determined by trypan blue (0.2 w/v) exclusion test. Cells were suspended at a density of 10^6^ cells mL^−1^ in round bottomed flasks rotating in a water bath maintained at 37°C in Krebs-Henseleit buffer (pH 7.4), supplemented with 12.5 mM HEPES under an atmosphere of 10% O_2_, 85% N_2_, and 5% CO_2_. Each flask contained 10 mL of hepatocyte suspension.

HCC hepatocytes and non-HCC hepatocytes immediately isolation the average viability was 90% and 92% for HCC and non-HCC hepatocytes by flow cytometry, respectively [15].

### 2.9. Isolation of Mitochondria from Rat Hepatocytes

Preparation of isolated rat liver cells is usually performed using the two-step collagenase liver perfusion technique of [16]. In order to evaluate cellular integrity (or viability), the trypan blue exclusion test is performed. Mitochondria are prepared from hepatocytes (30 × 10^6^ cells); 1 × 10^6^ cells/mL are resuspended in Krebs-Henseleit medium (pH 7.4) supplemented with 5 mM glucose, incubated under an atmosphere of 95% O_2_/5% CO_2_ in a shaking bath at 37°C for 2 h [17]. Cells are then pelleted (300 g for 3 min) and resuspended in 10 mL of Solution A (0.25 M sucrose, 0.01 M tricine, 1 mM EDTA, 10 mM NaH_2_PO_4_, 2 mM MgCl_2_, pH 8), supplemented with 0.4% BSA and frozen at –80°C for 10 min to break the plasma membrane, and centrifuged at 760 g for 5 min. The supernatant is kept while the pellet is homogenized, using Ultraturrax homogenizer for 10 min, followed by centrifugation at 760 g for 5 min. The supernatants from the previous two steps are combined and centrifuged for 20 min at 8000 g. Final mitochondrial pellets were suspended in Tris buffer containing (0.05 M Tris-HCl, 0.25 M sucrose, 20 mM KCl, 2.0 mM MgCl_2_, and 1.0 mM Na_2_HPO_4_, pH = 7.4) at 4°C, except for mitochondria used to assess ROS production, MMP, and swelling, which were suspended in respiration buffer (0.32 mM sucrose, 10 mM Tris, 20 mM Mops, 50 *μ*M EGTA, 0.5 mM MgCl_2_, 0.1 mM KH_2_PO_4_, and 5 mM sodium succinate), MMP assay buffer (220 mM sucrose, 68 mM D-mannitol, 10 mM KCl, 5 mM KH_2_PO_4_, 2 mM MgCl_2_, 50 *μ*M EGTA, 5 mM sodium succinate, 10 mM HEPES, 2 *μ*M Rotenone), and swelling buffer (70 mM sucrose, 230 mM mannitol, 3 mM HEPES, 2 mM Tris-phosphate, 5 mM succinate, and 1 *μ*M of rotenone). Protein concentrations were determined through the Coomassie blue protein-binding method as explained by Bradford [18]. The isolation of mitochondria was confirmed by the measurement of mitochondrial complex II (succinate dehydrogenase) activity [19]. Mitochondria were prepared fresh for each experiment and used within 1 h of incubation and all steps were strictly operated on ice to guarantee the isolation of high-quality mitochondria.

The concentrations of* D. kotschyi* (250, 500, and 1000 *μ*g/mL) were chosen based on our dose response study (data not shown) and mitochondrial samples were incubated in Tris buffer with different concentrations of* D. kotschyi* for 1 h.

### 2.10. Complex II Activity Assay Using MTT Test

The activity of mitochondrial complex II (succinate dehydrogenase) was assayed by measuring the reduction of MTT (3-[4,5-dimethylthiazol-2-yl]-2,5-diphenyltetrazoliumbromide). Briefly, 100 *μ*L of mitochondrial suspensions (0.5 mg protein/mL) was incubated with different concentrations of* D. kotschyi* (250, 500, and 1000 *μ*g/mL) at 37°C for 20 min; then, 0.4% of MTT was added to the medium and incubated at 37°C for 30 min. The product of formazan crystals was dissolved in 100 *μ*L DMSO and the absorbance at 570 nm was measured with an ELISA reader (Tecan, Rainbow Thermo, Austria) [20].

### 2.11. Determination of Mitochondrial ROS Level

The mitochondrial ROS measurement was performed using the fluorescent probe DCFH-DA. Briefly, isolated liver mitochondria were placed in respiration buffer containing 0.32 mM sucrose, 10 mM Tris, 20 mM Mops, 50 *μ*M EGTA, 0.5 mM MgCl_2_, 0.1 mM KH_2_PO_4_, and 5 mM sodium succinate. Following this step, DCFH-DA was added (final concentration, 10 *μ*M) to mitochondria and then incubated for 10 min at 37°C. Then, the fluorescence intensity of DCF was measured using Shimadzu RF-5000U fluorescence spectrophotometer at an excitation wavelength of 488 nm and emission wavelength of 527 nm [19].

### 2.12. Determination of the MMP

Mitochondrial uptake of the cationic fluorescent dye, Rhodamine 123, has been used for the estimation of mitochondrial membrane potential. The mitochondrial fractions (0.5 mg protein/mL) were incubated at 37°C with various concentrations of* D. kotschyi* and then 10 *μ*M of Rhodamine 123 was added to mitochondrial solution in MMP assay buffer (220 mM sucrose, 68 mM D-mannitol, 10 mM KCl, 5 mM KH_2_PO_4_, 2 mM MgCl_2_, 50 *μ*M EGTA, 5 mM sodium succinate, 10 mM HEPES, 2 *μ*M Rotenone). The fluorescence was monitored using Schimadzou RF-5000U fluorescence spectrophotometer at the excitation and emission wavelength of 490 nm and 535 nm, respectively [21].

### 2.13. Determination of Mitochondrial Swelling

Analysis of mitochondrial swelling after the isolated mitochondria (0.5 mg protein/mL) was estimated through changes in light scattering as monitored spectrophotometrically at 540 nm (30°C) as described [7]. Briefly, isolated mitochondria were suspended in swelling buffer (70 mM sucrose, 230 mM mannitol, 3 mM HEPES, 2 mM tris-phosphates, 5 mM succinate, and 1 *μ*M of rotenone) and incubated at 30°C with 250, 500, and 1000 *μ*g/mL of* D. kotschyi*. The absorbance was measured at 549 nm at 10 min time intervals with an ELISA reader (Tecan, Rainbow Thermo, Austria). A decrease in absorbance indicates an increase in mitochondrial swelling.

### 2.14. Determination of Cytochrome c Release

The concentration of cytochrome c was determined through using the Quantikine Rat/Mouse Cytochrome c Immunoassay kit provided by R & D Systems, Inc. (Minneapolis, Minn.). Briefly, a monoclonal antibody specific for rat/mouse cytochrome c was precoated onto the microplate. Seventy-five microliters of conjugate (containing monoclonal antibody specific for cytochrome c conjugated to horseradish peroxidase) and 50 *μ*L of standard and positive control were added to each well of the microplate. One microgram of protein from each supernatant fraction was added to the sample wells. All of the standards, controls, and samples were added to two wells of the microplate.

After 2 h of incubation, the substrate solution (100 *μ*L) was added to each well and incubated for 30 min. After 100 *μ*L of the stop solution was added to each well, the optical density of each well was determined by the aforementioned microplate spectrophotometer set to 450 nm.

### 2.15. Statistical Analysis

Results are presented as mean ± SD. All statistical analyses were performed using the SPSS software, version 17. Assays were performed 5 times and the mean was used for statistical analysis. Statistical significance was determined using the one-way ANOVA test, followed by the post hoc Tukey test. In some experiments, the two-way ANOVA test followed by the post hoc Bonferroni test was also performed. Statistical significance was set at *P* < 0.05.

## 3. Results

### 3.1. General Observation, Body and Liver Weights

During the entire study period, we observe any difference in food or water intake among the two experimental groups. Decrease apatite and food intake observed in group DEN/2-AAF as compare to control group rats.

**Figure 1:**
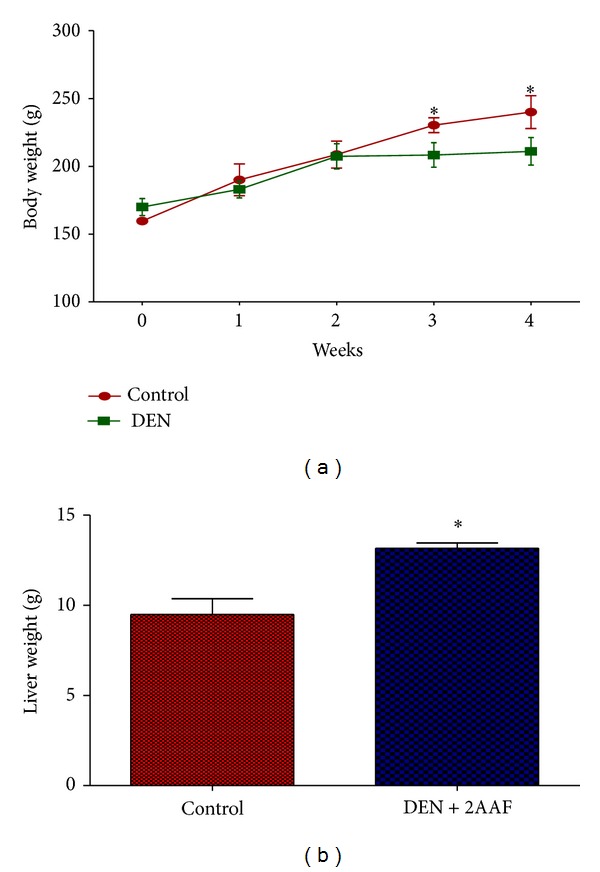
(a) The body weight of two animal groups following induction of hepatocarcinogenesis by DEN and 2-AAF. Significant (*P* < 0.05) differences in body weight were observed between DEN+2-AAF treated group and normal group at various time points. (b) The liver weight of two animal groups following induction of hepatocarcinogenesis by DEN and 2-AAF. Significant (*P* < 0.05) differences in body weight were observed between DEN+2-AAF treated group and normal group.

Average body weights of two animal groups at various time points were shown in Figure 1(a). The body weight of group II rats following DEN and 2-AAF treatment was significantly decreased (*P* < 0.001) compared with normal group I rats.

Average liver weight of group DEN/2-AAF was found to be significantly higher than that of control group (Figure 1(b)).

### 3.2. Serum Liver Enzymes and Hepatocarcinogenesis Marker

**Figure 2:**
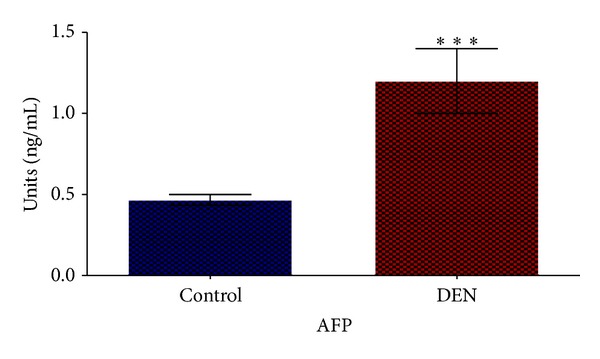
The levels of AFP (ng/mL) in the serum of control (I) and experimental (II) animal groups (*n* = 5 per group). Results are expressed as mean ± SD. Significant (*P* < 0.05) difference in AFP was observed between normal and DEN+2-AAF rat groups.

**Figure 3:**
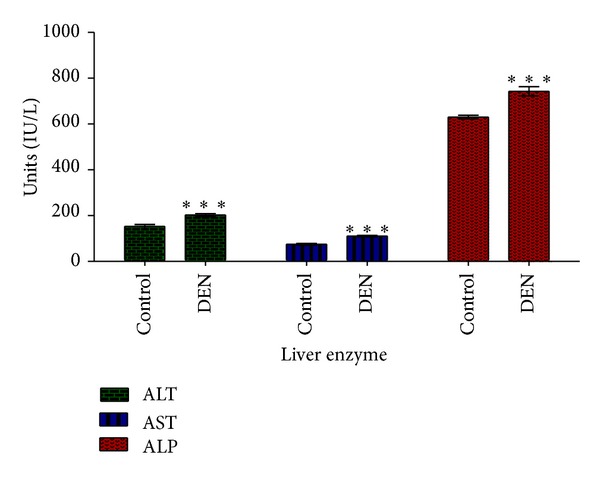
The activity of AST, ALT, and ALP (IU/L) in the liver of control (I) and experimental (II) groups (*n* = 5 rats). Results are expressed as mean ± SD. Significant (*P* < 0.05) difference in AST, ALT, and ALP was observed between normal and DEN+2-AAF rat groups.

The significant increase in the levels of hepatocarcinogenesis serum marker (AFP) was observed in group II compared with control group I (Figure 2). Rats treated by DEN and 2-AAF to develop hepatocarcinogenesis also showed significant increase (*P* < 0.05) in serum ALT, AST, and ALP concentrations (Figure 3).

### 3.3. Effect of In Vitro* D. kotschyi* Treatment on Mitochondrial Membrane Potential

**Figure 4:**
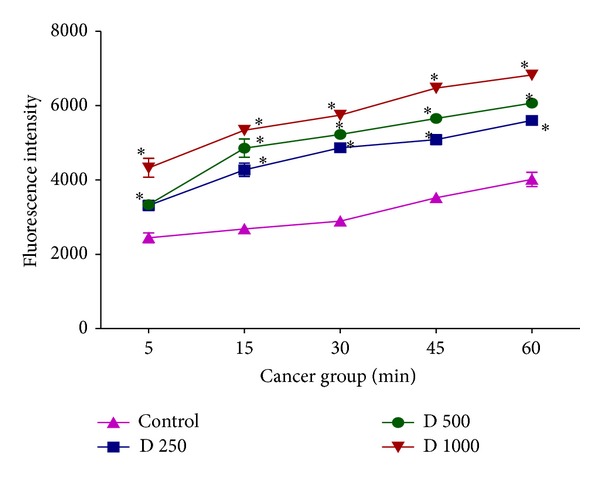
The effect of* D. kotschyi* extract on the mitochondrial membrane potential (MMP) in the liver mitochondria obtained from DEN/2-AAF treated rat group. MMP was measured by Rhodamine 123 as described in Section 2. Values presented as mean ± SD (*n* = 3).  *Minimal significance level *P* < 0.05.

**Figure 5:**
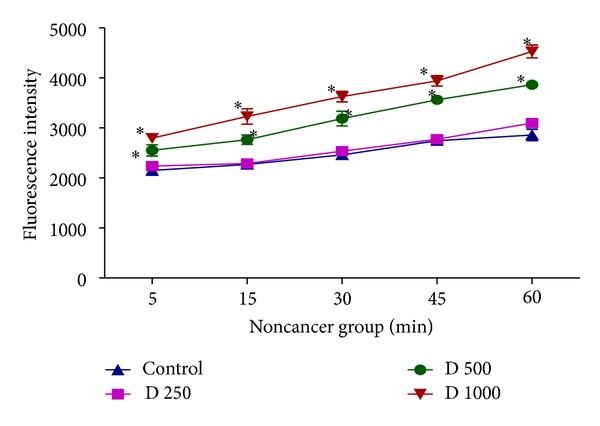
The effect of* D. kotschyi* extract on the mitochondrial membrane potential (MMP) in the liver mitochondria obtained from control rat group. MMP was measured by Rhodamine 123 as described in Section 2. Values presented as mean ± SD (*n* = 3).  *Minimal significance level *P* < 0.05.

MMP is a highly sensitive indicator of the mitochondrial permeability transition; therefore, the effect of* D. kotschyi* on MMP was measured by Rhodamine 123 staining. As shown in Figure 4,* D. kotschyi* extract concentrations (250, 500, and 1000 *μ*g/mL) significantly decreased the MMP in a time-related manner (*P* > 0.05) in mitochondria obtained from diethylnitrosamine/2-acetylaminofluorene-treated group. On the other hand, only 500 and 1000 *μ*g/mL concentrations but not 250 *μ*g/mL concentration of* D. kotschyi* extract significantly decreased the MMP following 60 min of incubation in the mitochondria isolated from rat liver of control group (Figure 5). Quenching of Rhodamine is proportional to the potential, so that an increase in fluorescence means depolarization. So, as shown in Figure 4, MMP and fluorescence intensity reported in our experiment are inversely proportional (see Tables 2 and 3).

### 3.4. Effect of* kotschyi* Treatment on Mitochondrial ROS Production

**Figure 6:**
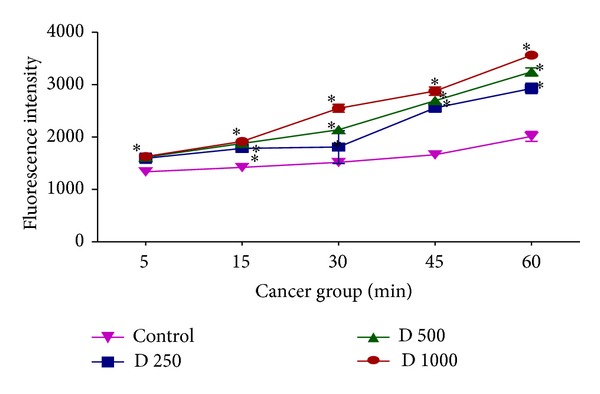
Increased ROS formation after addition of various concentrations of* D. kotschyi* extract (250, 500, and 1000 *µ*g/mL) at different time intervals within 60 min of incubation in the liver mitochondria obtained from DEN/2-AAF-treated rat group. Values presented as mean ± SD (*n* = 5).  *Minimal significance level *P* < 0.05.

**Figure 7:**
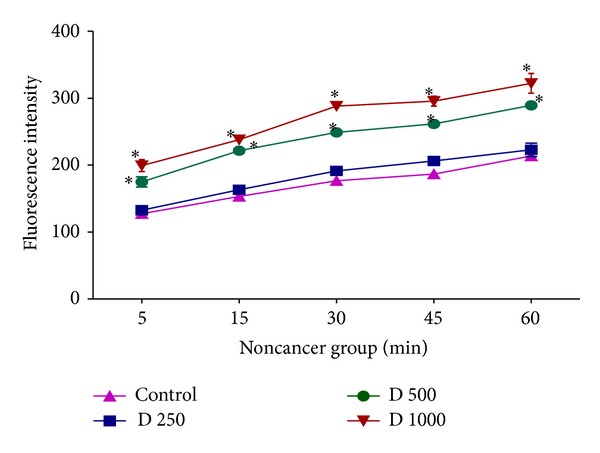
Increased ROS formation after addition of various concentrations of* D. kotschyi* extract (500 and 1000 *µ*g/mL) at different time intervals within 60 min of incubation in the liver mitochondria obtained from control rat group. Values presented as mean ± SD (*n* = 5).  *Minimal significance level *P* < 0.05.

As shown in Figure 6, different concentrations of* D. kotschyi* extract (250, 500, and 1000 *μ*g/mL) induced significant H_2_O_2_ formation (*P* > 0.05) in liver mitochondria obtained from DEN+2-AAF treated rat group in a time dependent manner. On the other hand, only 500 and 1000 *μ*g/mL concentrations but not 250 *μ*g/mL concentration of* D. kotschyi* extract induced significant H_2_O_2_ formation following 60 min of incubation in the mitochondria isolated from rat liver of control group (Figure 7) (see Tables 4 and 5).

### 3.5. Effect of* D. kotschyi* Extract on Mitochondrial Swelling

**Figure 8:**
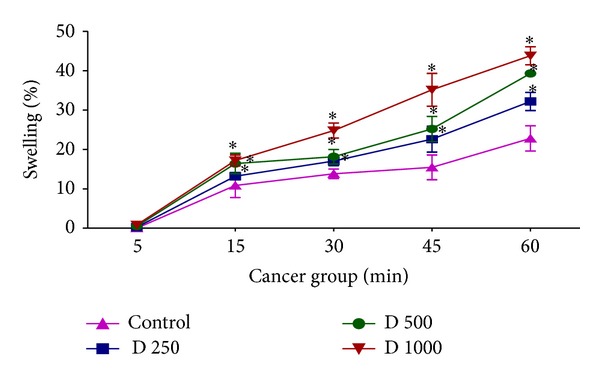
The effect of* D. kotschyi* extract on the mitochondrial swelling in the liver mitochondria obtained from DEN/2-AAF-treated rat group. Mitochondrial swelling was measured spectrophotometrically as described in Section 2. Values presented as mean ± SD (*n* = 5).  *Minimal significance level *P* < 0.05.

**Figure 9:**
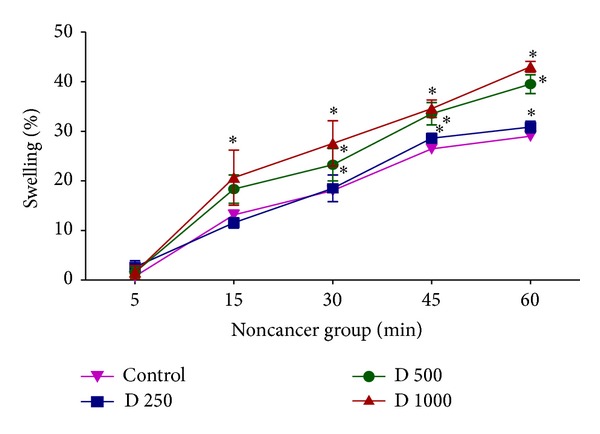
The effect of* D. kotschyi* extract on the mitochondrial swelling in the liver mitochondria obtained from control rat group. Mitochondrial swelling was measured spectrophotometrically as described in Section 2. Values presented as mean ± SD (*n* = 5).  *Minimal significance level *P* < 0.05.

Moreover, we monitored the decrease of absorbance of mitochondrial samples at 540 nm to assay mitochondrial swelling, another indicator of mitochondrial membrane permeability transition. Addition of different concentrations of* D. kotschyi* extract (250, 500, and 1000 *μ*g/mL) to mitochondrial suspensions obtained from livers of diethylnitrosamine/2-acetylaminofluorene-treated rat group led to significant mitochondrial swelling in a time-dependent manner (*P* < 0.05) (Figure 8). On the other hand only 500 and 1000 *μ*g/mL concentrations but not 250 *μ*g/mL concentration of* D. kotschyi* extract induced significant mitochondrial swelling (*P* < 0.05) following 60 min of incubation in the mitochondria isolated from rat liver of control group (Figure 9) (see Tables 6 and 7).

### 3.6. Effect of* D. kotschyi* Extract on Cytochrome c Release

**Figure 10:**
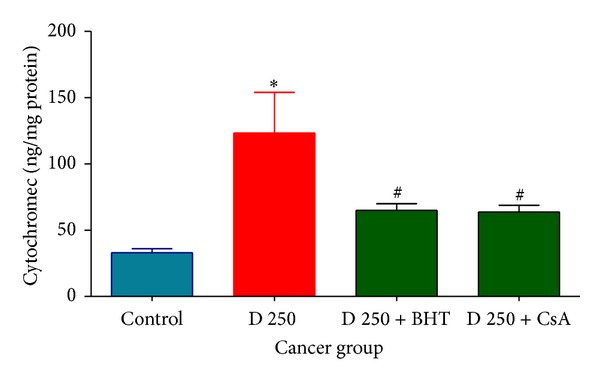
Effect of* D. kotschyi* (250 *µ*g/mL) on the cytochrome c release in the liver mitochondria isolated from DEN+2-AAF treated rat group. As shown in this figure, pretreatment of with BHT or CsA significantly inhibited cytochrome c release in the cancerous liver mitochondria. The amount of expelled cytochrome c from mitochondrial fraction into the suspension buffer was determined using Rat/Mouse Cytochrome c ELISA kit as described in Section 2. Values presented as mean ± SD (*n* = 5).  ^∗#^Minimal significance level *P* < 0.05.

**Figure 11:**
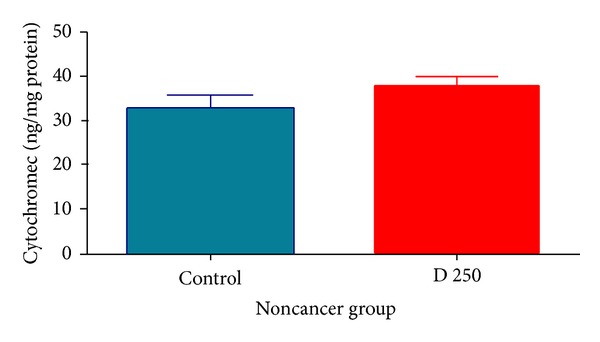
Effect of* D. kotschyi* extract (250 *µ*g/mL) on the cytochrome c release in the liver mitochondria isolated from control rat group. The amount of expelled cytochrome c from mitochondrial fraction into the suspension buffer was determined using Rat/Mouse Cytochrome c ELISA kit as described in Section 2. Values presented as means (*n* = 5). As shown in this figure,* D. kotschyi* extract (250 *µ*g/mL) did not induce significant cytochrome c release in the liver mitochondria isolated from control rat group. Minimal significance level was *P* < 0.05.

Our results showed that D. kotschyi extract significantly caused collapse of the mitochondrial membrane potential and mitochondrial swelling. These events could result from mitochondrial permeability transition and release of cytochrome c from mitochondria into the cytosolic fraction. As shown in Figures 10 and 11,* D. kotschyi* extract (250 *μ*g/mL) induced significant (*P* < 0.05) release of cytochrome c on the liver mitochondria isolated from DEN+2-AAF treated but not control rat group. Significantly, the pretreatment of* D. kotschyi*-treated mitochondria with the MPT inhibitor of cyclosporine A (CsA) and butylated hydroxyl toluene (BHT), an antioxidant, inhibited cytochrome c release as compared with* D. kotschyi*-treated group (250 *μ*g/mL) (*P* < 0.05), indicating the role of oxidative stress and MPT pore opening in cytochrome c release.

## 4. Discussion

Cancer is a growing health problem around the world and is the second leading cause of death after heart disease [22]. According to a recent report by the World Health Organization (WHO), from a total of 58 million deaths worldwide in 2008, cancer accounted for 13% [23]. HCC is the third leading cause of cancer-related death worldwide. The incidence of this disease is increasing and it is one of the key indications for liver transplantation. Chronic infection with hepatitis B virus is the leading cause of HCC, closely followed by infection with hepatitis C virus [24]. Other factors contributing to the development of HCC include alcoholism and obesity [24]. Although treatment options have improved in the past 30 years, particularly with the approval of several molecular-targeted therapies, prognosis remains dismal for many patients. Advances are being made in understanding the mechanisms underlying HCC, which in turn could lead to novel therapeutics, but more progress is urgently needed in this area [24]. Novel natural products offer opportunities for innovation in drug discovery [25]. In fact, natural products play a major role in cancer prevention and treatment. A considerable number of antitumor agents currently used in the clinic are of natural origin [26]. Among them, plants have been the chief source of natural compounds used for medicine. During the 1960s, the National Cancer Institute (United States) began to screen plant extracts with antitumor activity [27]. Natural compounds isolated from medicinal plants, as rich sources of novel anticancer drugs, have been of increasing interest since then [25]. During long-term folk practice, a large number of anticancer medicinal herbs and many relevant prescriptions have been screened and used for treating and preventing various cancers [28]. The* D. kotschyi* is a plant belonging to the family* Labiatae* and is found abundantly in south western Asia.* D. kotschyi* has been used as a medicinal herb for several years in Iran folk medicine due to its antispasmodic and analgesic, antihyperlipidemic [5] and immunomodulatory effects [6].

Mitochondria are semiautonomous subcellular organelles that play essential roles in cellular metabolism and programmed cell death pathways. Genomic, functional, and structural mitochondrial alterations have been associated with cancer. Due to the specific alterations that occur in cancer cell mitochondria, these organelles may provide promising targets for cancer therapy [29].

Studies dealing with the morphology of tumoral mitochondria have shown, at least in rapidly growing tumors, that the mitochondria are fewer, smaller, and morphologically altered. It has also been observed that mitochondria from liver tumors are more fragile than normal liver mitochondria. In addition, a number of biochemical differences have been observed between tumoral and normal liver mitochondria. As a result of early observations by Warburg that transformed cells displayed an extremely high aerobic glycolysis, a great number of studies have looked for defects in the enzymes involved in electron transport, oxidative phosphorylation, and other mitochondrial specific functions. Most of these studies indicated that the tumor cell content of many mitochondrial enzymes include *β*-hydroxybutyrate dehydrogenase, malate dehydrogenase, adenylate kinase, monoamine oxidase, rotenone-insensitive NADH-cytochrome c reductase, succinate dehydrogenase, and cytochrome oxidase was severely depressed when compared to normal liver [30].

In this study we induced HCC by DEN as carcinogen and 2-AAF as a promoter in Wistar rats. The body weight of each rat was recorded (Figure 1) before blood was collected by cardiac puncture. Decreased body weight and increased serum AFP (Figure 2) concentration, ALT, AST, and ALP activity (Figure 3) confirmed induction of hepatocellular carcinoma in DEN+2-AAF treated rat group. The isolated hepatocytes obtained from livers of DEN+2-AAF treated rat group were then sorted by flow cytometry to obtain pure HCC hepatocytes. Different concentrations of* D. kotschyi* extract (250, 500, and 1000 *μ*g/mL) significantly decreased the MMP in the mitochondria of HCC hepatocytes in a time-related manner (Figure 4). However, 250 *μ*g/mL concentration of* D. kotschyi* extract showed no effect on MMP of liver mitochondria isolated from control rat group within 60 min of incubation (Figure 5). Different concentrations of* D. kotschyi* extract (250, 500, and 1000 *μ*g/mL) induced significant elevation on H_2_O_2_ production in the mitochondria of HCC hepatocytes obtained from DEN+2-AAF treated rat group in a time dependent manner (Figure 6). As shown in Figure 7, again 250 *μ*g/mL concentration of* D. kotschyi* extract showed no effect on H_2_O_2_ production in the liver mitochondria obtained from non-HCC control rat group during 60 min of incubation (*P* < 0.05). The change of mitochondrial swelling as an indicator of mitochondrial permeability transition was monitored.* D. kotschyi* extract (250, 500, and 1000 *μ*g/mL) induced significant mitochondrial swelling in the tumoral mitochondria obtained from DEN+2-AAF treated rat group (Figure 8). However, 250 *μ*g/mL concentration of* D. kotschyi* showed no significant effect (*P* < 0.05) on mitochondrial swelling in the liver mitochondria obtained from non-HCC control rat group during 60 min of incubation (*P* < 0.05) (Figure 9). Finally* D. kotschyi* extract (250 *μ*g/mL) significantly caused the cytochrome c release and disruption of mitochondrial outer membrane integrity in the liver mitochondria obtained from DEN+2-AAF treated but not non-HCC control rat group during 60 min of incubation (*P* < 0.05) (Figures 10 and 11).

A novel chemotherapeutic approach has been explored in recent years. This approach is based on cytotoxic molecules that induce direct perturbation of mitochondria in only tumoral but not nontumoral normal cells, thereby circumventing upstream proapoptotic pathways that may be mutated or lacking in the cancer cell. Many of these cytotoxic molecules act directly on the mitochondria permeability transition (MPT) pores and in some cases by increasing ROS production by disruption in mitochondrial electron transport chain and increasing Ca^+2^ in matrix of mitochondria leading to mitochondrial swelling, membrane permeability transition, and finally release of cytochrome c and other apoptogenic proteins from the mitochondria and formation of the caspase-3 activation complex, the apoptosome in the cytosol of tumoral cell [31, 32].

It is likely that extract of* D. kotschyi* contains natural products which can have effect on mitochondria through production of ROS that leads to opening of mitochondrial MPT pores and consequent collapse of the inner membrane potential and osmotic swelling of the mitochondrial matrix. Opening of MPT pores could release cytochrome c and other apoptogenic proteins from the mitochondria subsequently and start the apoptosis signaling through formation of the caspase-3 activation complex, the apoptosome in cancerous liver hepatocytes. The golden point in our findings is that (250 *μ*g/mL) concentration of* D. kotschyi* extract significantly caused H_2_O_2_ production, collapse of mitochondrial membrane potential, mitochondrial swelling, and cytochrome c release or disruption of mitochondrial outer membrane integrity in the liver mitochondria obtained from DEN+2-AAF treated tumoral but not nontumoral control rat group during 60 min of incubation (*P* < 0.05) which proposes* Dracocephalum kotschyi* as a promising anticancer plant acting on liver cancer cell mitochondria.

Accordingly, both dissipation of mitochondrial membrane potential and cytochrome c release are the important indicators of cell apoptosis and important endpoints for the determination of mitochondrial dysfunction. As manifested through the results,* D. kotschyi* caused significant expulsion of cytochrome c from mitochondria. Moreover, Cs A and BHT pretreatment completely blocked the* D. kotschyi*-induced release of cytochrome c from the mitochondria which supports the hypothesis that the apoptosis induction via* D. kotschyi* is due to an oxidative stress and depends on the opening of the mitochondrial transition pore.

## Figures and Tables

**Table 1:** 

Flavonoid	Amount (*µ*g/g)
Luteolin	80 ± 0.01
Naringenin	10 ± 0.003
Apigenin	100 ± 0.01
Isokaempferoide	30 ± 0.009
Cirsimaritin	20 ± 0.003
Penduletin	20 ± 0.005
Xanthomicrol	90 ± 0.01
Calycoperin	60 ± 0.01

Total	0.05% (w/w) of dry sample

Results presented as mean values ± SD of three independent experiments.

**Table 2:** The effect of different concentrations of *D. kotschyi* extract on the mitochondrial membrane potential (MMP) in the liver mitochondria obtained from DEN/2-AAF-treated rat group.

60 min	45 min	30 min	15 min	5 min	Group
4014 ± 333	3520 ± 161	2893 ± 80	2682 ± 35	2444 ± 227	Control
5602 ± 159∗	5085 ± 234∗	4866 ± 152∗	4273 ± 306∗	3315 ± 234∗	D 250 *µ*g/mL
6066 ± 123∗	5654 ± 104∗	5226 ± 95∗	4522 ± 159∗	3338 ± 66∗	D 500 *µ*g/mL
6824 ± 28∗	6473 ± 84∗	5745 ± 119∗	5337 ± 211∗	4326 ± 440∗	D 1000 *µ*g/mL

Values presented as mean ± SD (*n* = 5). ∗Minimal significance level *P* < 0.05.

**Table 3:** The effect of different concentrations of *D. kotschyi* extract on the mitochondrial membrane potential (MMP) in the liver mitochondria obtained from control rat group.

60 min	45 min	30 min	15 min	5 min	Group
2859 ± 197	2744 ± 107	2462 ± 65	2270 ± 94	2150 ± 161	Control
3098 ± 88	2775 ± 87	2536 ± 36	2289 ± 63	2237 ± 90	D 250 *µ*g/mL
3864 ± 133∗	3563 ± 118∗	3186 ± 254∗	2766 ± 162∗	2552 ± 195∗	D 500 *µ*g/mL
4528 ± 223∗	3942 ± 178∗	3629 ± 181∗	3230 ± 266∗	2797 ± 85∗	D 1000 *µ*g/mL

Values presented as mean ± SD (*n* = 5). ∗Minimal significance level *P* < 0.05.

**Table 4:** The effect of different concentrations of *D. kotschyi* extract on the ROS formation in the liver mitochondria obtained from DEN/2-AAF-treated rat group.

60 min	45 min	30 min	15 min	5 min	Group
2010 ± 162	1661 ± 45	1516 ± 82	1420 ± 24	1336 ± 13	Control
2926 ± 166∗	2556 ± 139∗	1808 ± 544∗	1782 ± 129∗	1594 ± 160∗	D 250 *µ*g/mL
3241 ± 129∗	2692 ± 100∗	2137 ± 96∗	1875 ± 120∗	1614 ± 80∗	D 500 *µ*g/mL
3560 ± 111∗	2875 ± 137∗	2547 ± 131∗	3230 ± 101∗	1626 ± 119∗	D 1000 *µ*g/mL

Values presented as mean ± SD (*n* = 5). ∗Minimal significance level *P* < 0.05.

**Table 5:** The effect of different concentrations of *D. kotschyi* extract on the ROS formation in the liver mitochondria obtained from control rat group.

60 min	45 min	30 min	15 min	5 min	Group
213 ± 1	186 ± 3	176 ± 4	153 ± 5	127 ± 4	Control
223 ± 17	206 ± 3	191 ± 2	163 ± 4	132 ± 3	D 250 *µ*g/mL
289 ± 7∗	261 ± 2∗	249 ± 2∗	221 ± 5∗	175 ± 13∗	D 500 *µ*g/mL
322 ± 25∗	295 ± 12∗	288 ± 11∗	238 ± 7∗	199 ± 15∗	D 1000 *µ*g/mL

Values presented as mean ± SD (*n* = 5). ∗Minimal significance level *P* < 0.05.

**Table 6:** The effect of *D. kotschyi* extract on the mitochondrial swelling in the liver mitochondria obtained from DEN/2-AAF treated rat group.

60 min	45 min	30 min	15 min	5 min	Group
22.8 ± 4	15.4 ± 4	13.8 ± 1.6	10.8 ± 4	0	Control
32.2 ± 3.2∗	22.6 ± 4.6∗	17 ± 1.6∗	13.2 ± 1.2∗	0.25 ± 0.35	D 250 *µ*g/mL
39.3 ± 0.56∗	25.2 ± 4.5∗	18.15 ± 2.6∗	16.4 ± 3.6∗	0.5 ± 0.7	D 500 *µ*g/mL
43.8 ± 3.2∗	35.1 ± 5.8∗	24.8 ± 2.6∗	17.2 ± 1.9∗	0.85 ± 1.2	D 1000 *µ*g/mL

Values presented as mean ± SD (*n* = 5). ∗Minimal significance level *P* < 0.05.

**Table 7:** The effect of *D. kotschyi* extract on the mitochondrial swelling in the liver mitochondria obtained from control group.

60 min	45 min	30 min	15 min	5 min	Group
29 ± 0.56	26.45 ± 0.9	18 ± 0.35	13.15 ± 1.3	0.75 ± 1	Control
30.85 ± 1.62	28.6 ± 1.41	18.5 ± 3.81	11.55 ± 1.48	2.65 ± 1.76	D 250 *µ*g/mL
39.5 ± 2.6∗	33.55 ± 3.18∗	23.25 ± 4∗	23.25 ± 4	1.55 ± 1.48	D 500 *µ*g/mL
43 ± 1.55∗	34.55 ± 2.4∗	27.55 ± 6.4∗	20.6 ± 7.8∗	1.5 ± 1	D 1000 *µ*g/mL

Values presented as mean ± SD (*n* = 5). ∗Minimal significance level *P* < 0.05.
